# Modulating Human Auditory Processing by Transcranial Electrical Stimulation

**DOI:** 10.3389/fncel.2016.00053

**Published:** 2016-03-07

**Authors:** Kai Heimrath, Marina Fiene, Katharina S. Rufener, Tino Zaehle

**Affiliations:** Department of Neurology, Otto-von-Guericke University MagdeburgMagdeburg, Germany

**Keywords:** transcranial direct current stimulation, transcranial alternating current stimulation, transcranial random noise stimulation, auditory processing

## Abstract

Transcranial electrical stimulation (tES) has become a valuable research tool for the investigation of neurophysiological processes underlying human action and cognition. In recent years, striking evidence for the neuromodulatory effects of transcranial direct current stimulation, transcranial alternating current stimulation, and transcranial random noise stimulation has emerged. While the wealth of knowledge has been gained about tES in the motor domain and, to a lesser extent, about its ability to modulate human cognition, surprisingly little is known about its impact on perceptual processing, particularly in the auditory domain. Moreover, while only a few studies systematically investigated the impact of auditory tES, it has already been applied in a large number of clinical trials, leading to a remarkable imbalance between basic and clinical research on auditory tES. Here, we review the state of the art of tES application in the auditory domain focussing on the impact of neuromodulation on acoustic perception and its potential for clinical application in the treatment of auditory related disorders.

## Introduction

During the last decades transcranial electrical stimulation (tES) has been revived as a technique to directly influence brain activity and its related alterations in behavior (Fox, 2011). Although tES has a long history, the mode of action, application, and relevance for neuroscientific and clinical purposes are controversially discussed and still require systematic investigation.

The advent of functional brain imaging has extended our knowledge about specific neural mechanisms involved in cognitive, motor, and perceptual processes. However, neuroimaging results are inherently correlational showing that activity in specific brain areas is associated with certain perceptions and behaviors. Accordingly, inferences of causality cannot be drawn from imaging studies. Neurostimulation enables us to modulate the excitability of brain areas and to observe the effect on behavior. Therefore, we can utilize tES to make causal inferences about the relationship between areas of the brain and behavior. Thus, tES now opens new strategies for testing hypotheses on the causal relation of cortical reactivity and function (Fox, 2011; Miniussi and Ruzzoli, 2013; Filmer et al., 2014).

However, while the majority of human tES studies have focused on the motor domain (Jaberzadeh and Zoghi, 2013), human cognition (Antal et al., 2014), and to a lesser extent on the modulation of visual (Antal et al., 2011) and somatosensory perception (Feurra et al., 2011; Costa et al., 2015), surprisingly little is known about the impact of tES on auditory processing. Evidence of behavioral and direct neurophysiological consequences of tES in the auditory domain is rather sparse. Moreover, while only a few studies systematically investigated the impact of auditory tES so far, an overwhelming number of clinical trials already applied auditory tES as a treatment in several patient groups, leading to an imbalance between basic and clinical research on tES of the auditory system. As a consequence, clinical outcomes are heterogeneous and the nature of the neurophysiological mechanisms underlying the tES-related clinical alterations is not yet well understood.

In the following we review current results of tES of the auditory system. For that purpose PubMed online database was searched. The following keywords were used and combined to select the most relevant articles: transcranial direct current stimulation (tDCS), transcranial alternating current stimulation (tACS), transcranial random noise stimulation (tRNS) combined with auditory processing, tinnitus, or aphasia. All studies had to be conducted in humans. In the present review we introduce tDCS, tACS, and tRNS and summarize studies investigating their effects on auditory processing. We specifically emphasize the presumed mechanism of action during and after stimulation as well as the impact of different stimulation parameters on behavioral and neurophysiological outcome. Finally, we discuss future challenges in tES and the significance of recent findings for clinical application.

### Transcranial Direct Current Stimulation

In cognitive research and clinical application, tDCS is probably the most frequently used non-invasive tES method that delivers low currents to the cerebral cortex resulting in the modulation of cortical excitability (Nitsche et al., 2003b, 2008). The tDCS current flows between an active and a reference electrode. While a part of this current is shunted through the scalp, the majority is delivered to the brain tissue (Miranda et al., 2006; Neuling et al., 2012b), thereby inducing diminutions or enhancements of cortical excitability (Nitsche et al., 2008). The direction of the tDCS-induced effect depends on the current polarity. Anodal tDCS typically has an excitatory effect while cathodal tDCS decreases the cortical excitability in the region under the electrode (Nitsche and Paulus, 2000; Nitsche et al., 2003b). Specifically, anodal tDCS causes a depolarization of the resting membrane potential and increases the firing rate of the neurons, whereas cathodal tDCS decreases the firing rate via hyperpolarization of the resting membrane potential (Bindman et al., 1962; Purpura et al., 1965).

The effects of tDCS are not limited to modulations of cortical excitability during stimulation (online effect), but outlast the stimulation period by several minutes or hours (Bindman et al., 1962, 1964; Nitsche and Paulus, 2000, 2001). This aftereffect or offline effect of tDCS relies on long-term synaptic changes associated with long-term potentiation (LTP) and long-term depression (LTD). Specifically, tDCS induced post-synaptic polarization is caused by altered pre-synaptic input due to changed firing rates which leads to enhanced *N*-methyl-D-aspartate (NMDA) receptor-efficiency resulting in an increase of the intracellular Ca^2+^ level. While anodal aftereffects are suggested to induce LTP due to enhanced firing rate, cathodal tDCS reduces firing rate followed by LTD (Liebetanz et al., 2002; Nitsche et al., 2002, 2003a; Stagg and Nitsche, 2011; Monte-Silva et al., 2013).

These differences underlying the physiological actions of online vs. offline tDCS can also lead to contradictive effects of tDCS, with enhanced motor as well as cognitive performance during and opposite effects after the tDCS application (Kuo et al., 2008; Stagg et al., 2011; Martin et al., 2014). Such opposite online vs. offline effects have also been demonstrated for the visual domain with improved perceptual learning after but not during cathodal tDCS (Pirulli et al., 2013). Accordingly, tDCS efficiency on cortical excitability critically depends on the timing of the stimulation. Also opposing results regarding the heuristic anodal-exciting vs. cathodal-inhibiting dichotomy have been reported. Generally, as mentioned above, it is assumed that anodal tDCS has an excitatory effect whereas cathodal tDCS has an inhibitory effect on the local cerebral cortex under the electrode. However, several recent data demonstrate an opposite anodal/cathodal dichotomy, with, e.g., decreased reactivity of specific brain regions after anodal (Chen et al., 2013) and increased reactivity after cathodal stimulation (Zaehle et al., 2011). This, on a first glance, unintuitive effect that an artificially enhanced neural excitability does not increase performance *per se* has been related to a non-linear relationship between stimulation effect and resulting behavior. In an optimal and unaffected level of neuronal reactivity, both an increase as well as a decrease of neuronal reactivity will deteriorate the processing of this cortical area (Yerkes and Dodson, 1908; Baldi and Bucherelli, 2005). Such an inverted U-shape relation has been demonstrated for the influences of auditory tDCS on acoustic processing (Heimrath et al., 2014) and for the influence of psychotropic drugs on tDCS effects (Monte-Silva et al., 2013). Recently, Krause et al. (2013) captured this schema by taking into account the excitation/inhibition (E/I) balance measured by the ratio of glutamate/GABA. They proposed that only a balanced E/I ratio leads to an optimal level of processing efficiency. Anodal and cathodal tDCS may shift the balance due to changes of glutamate/GABA concentration (Filmer et al., 2014). Thus, while anodal tDCS causes an over-activation resulting in decreased performance, cathodal tDCS can improve performance by restoring the optimum in subjects with non-optimal E/I level.

Accordingly, the assumption that polarity-specific changes of cortical excitability are simply reflected in behavioral effects is rather ambiguous (Jacobson et al., 2012; Miniussi and Ruzzoli, 2013). Therefore, the acquisition of direct electrophysiological data, e.g., by recording the EEG during and after the application of tDCS may help to assess the underlying physiological basis and thereby to improve the efficiency of auditory tDCS schemas as well as to identify the actual brain–behavior relationship by causal inferences (Sale et al., 2015).

### Transcranial Alternating Current Stimulation/Transcranial Random Noise Stimulation

Besides the application of constant current, alternating current can also be applied transcranially to the human brain. While during tACS sinusoidal currents at single frequencies are applied, a multitude of sinusoidal oscillations with various different frequencies is used during tRNS.

Generally, tACS synchronizes cortical oscillations by inducing distinct frequency patterns (Antal and Paulus, 2013; Herrmann et al., 2013b). This approach enables us to study the influence of specific neuronal oscillations on perceptual (Kanai et al., 2010; Neuling et al., 2012a; Brignani et al., 2013; Helfrich et al., 2014) and cognitive processes (Marshall et al., 2006; Polania et al., 2012; Janik et al., 2015). It has been suggested that the external periodic stimulation by tACS directly entrains underlying brain oscillations causing a temporal alignment of intrinsic brain activity to the externally applied alternating current (Herrmann et al., 2013a,b). This entrainment involves both, the oscillatory frequency as well as the phase angle of the neural population to the external driving source. While tDCS modulates the general reactivity of the stimulated cortical region, tACS influences the information transfer within and between cortical regions. Besides the frequency and the phase, the applied intensity shapes the direction and duration of the tACS-effect, too. Of note, various tACS-studies adjust the stimulation intensity individually below the subjects’ sensation threshold, while in tDCS-studies the intensity is usually equal for all subjects. In addition to the online effects of tACS via entrainment, aftereffects extending the stimulation period by several minutes have also been demonstrated (Veniero et al., 2015). This offline effect has been related to synaptic changes via spike-timing dependent plasticity (Zaehle et al., 2010; Polania et al., 2012; Vossen et al., 2015).

**FIGURE 1:**
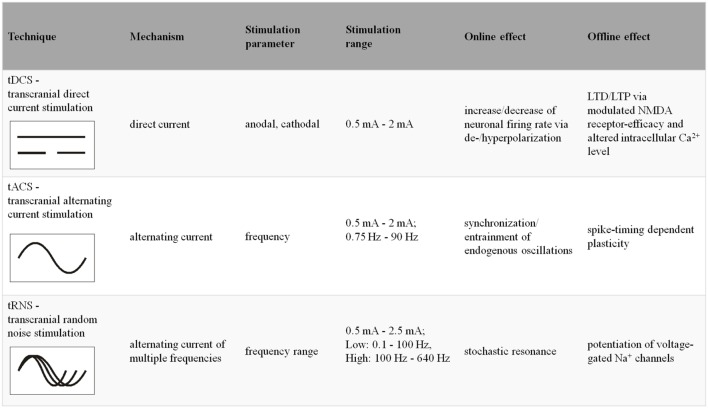
**Stimulation parameters and underlying neurophysiological mechanisms of the individual tES-techniques (LTD, long-term depression; LTP, long-term potentiation; NMDA, *N*-methyl-D-aspartate)**.

Finally, tRNS induces noise by means of simultaneously applied alternating currents of different frequencies and amplitudes into a plastic system (i.e., the cortex region). Via stochastic resonance, the applied noise increases the dominant and functionally relevant oscillatory response in the system (Longtin, 1993; McDonnell and Abbott, 2009). Since it has been shown that the efficacy of tACS to entrain and thus to affect endogenous oscillations is most pronounced when the frequency of the stimulation coincides with the dominant frequency of the target region, tRNS seems capable of taking inter- and intraindividual differences of the endogenous oscillatory frequency into account (for a schematic overview on individual techniques see **Figure 1**).

## Effects of tDCS on Neuronal Reactivity in the Auditory System

Neuromodulatory changes induced by tDCS have been successfully demonstrated in the motor (Priori et al., 1998; Nitsche and Paulus, 2000; Sehm et al., 2013b), visual (Antal et al., 2003; Accornero et al., 2007; Peters et al., 2013), and somatosensory system (Dieckhofer et al., 2006; Antal et al., 2008; Sehm et al., 2013a) as well as in the cognitive domain (Heimrath et al., 2012; Santiesteban et al., 2012; Floel, 2014). In the auditory domain, however, evidence for tDCS induced alterations of the auditory cortex (AC) reactivity and related behavioral changes is still sparse. Notwithstanding, a number of studies demonstrated the effectiveness of tDCS to alter cortical reactivity of the AC as well as its related auditory perceptual processing. Importantly and analogously to other domains, the majority of reports based their evidence on the investigation of behavioral changes associated with tDCS whereas only a minority of studies also elucidated direct electrophysiological consequences of effective tDCS modulations of the human AC.

In a first approach we investigated cortical reactivity of the human AC after anodal and cathodal tDCS (Zaehle et al., 2011). For this purpose we placed active tDCS electrodes over a temporal or a temporo-parietal location and a reference electrode over the contralateral supraorbital area. Each of our participants performed four consecutive sessions at 1-week intervals and received in two out of four sessions tDCS of the primary AC, while in the two remaining sessions, tDC-stimulation was applied over a secondary auditory region. The session order was counterbalanced across participants. Furthermore, in each session, participants underwent one sham, and one verum stimulation with the sham condition always preceding the verum stimulation condition to avoid carryover effects of tDCS. After receiving tDCS we recorded auditory evoked potentials (AEPs) in response to sinusoidal tones of 1 kHz and found tDCS-induced modulations of auditory evoked brain activity as a function of stimulation site and condition (offline effect). Both, anodal and cathodal stimulation over the primary and secondary AC affected sensory acoustic processing. Consequently, by revealing polarity-specific effects of anodal and cathodal tDCS on AC reactivity, we demonstrated for the first time, that the excitability of the AC can be directly modulated by tDCS. However, while anodal tDCS over the temporal lobe increased the P50 amplitude, cathodal stimulation over the temporo-parietal area (TPA) increased the N1 component of the AEP (Zaehle et al., 2011). Besides these direct electrophysiological evidences for tDCS-related alterations of the human AC, several further studies reported effects of auditory tDCS on different aspects of acoustic perception (for specific stimulation parameters and outcome measures see **Table 1**).

**Table 1:** General parameters and results of transcranial direct current stimulation (tDCS) studies.

Reference	Sample size	Stimulation electrode position and size	Reference electrode position and size	Stimulation parameters	Stimulation types	Timepoint of data acquisition	Paradigm	Results
Chen et al., 2013	10	F4, 35 cm^2^	contralateral supraorbital, 35 cm^2^	2.0 mA for 25 min	Anodal cathodal sham	Offline	Oddball paradigm (spectral and temporal deviants)	Anodal tDCS decreased MMN amplitude to spectral deviants
Heimrath et al., 2014	15	T7, T8, 25 cm^2^	Contralateral C4, C3, 50 cm^2^	1.5 mA for 13 min	Anodal sham	Online	Between-channel gap detection task	Anodal tDCS over the left AC diminished temporal resolution
Heimrath et al., 2015	12	C5, C6, HD-tDCS ring electrodes (outer *r* = 12 mm; inner *r* = 6 mm)	FC5/FC6, C3/C4, CP5/CP6, T7/T8, HD-tDCS ring electrodes (outer *r* = 12 mm; inner *r* = 6 mm)	0.5 mA for 21 min	Anodal cathodal sham	Online	MMN paradigm (spectral and temporal deviants)	Anodal tDCS over the left AC increased MMN amplitude to temporal deviants
Heimrath et al., 2016	13	Simultaneous T7 and T8, 25 cm^2^	Longitudinally over Cz, 50 cm^2^	1.5 mA for 22 min	Anodal cathodal sham	Online and offline	Phonetic perception task	Cathodal tDCS improved phonetic categorization (online), anodal tDCS increased P50 (offline)
Impey and Knott, 2015	12	C5-T7, unspecified size	Contralateral forehead, unspecified size	2.0 mA for 20 min	Anodal sham	Offline	Oddball paradigm (spectral deviants)	Anodal tDCS over the left AC increased MMN amplitude to spectral deviants
Ladeira et al., 2011	11	Simultaneous T3 and T4, 35 cm^2^	Contralateral deltoid muscle, 35 cm^2^	2.0 mA for 10 min	Anodal cathodal sham	Online	Random gap detection test	Anodal tDCS enhanced temporal resolution, cathodal diminished temporal resolution
Loui et al., 2010	9	TP7-C5, TP8-C6, F7-C5, F8-C6, 16 cm^2^	Contralateral supraorbital, 16 cm^2^	2.0 mA for 20 min	Cathodal sham	Offline	Pitch matching task	Cathodal tDCS over the inferior frontal and superior temporal cortex decreased pitch discrimination
Mathys et al., 2010	21	C3-T3, C4-T4, 16.3 cm^2^	Contralateral supraorbital, 30 cm^2^	2.0 mA for 25 min	Cathodal anodal sham	Offline	Pitch discrimination task	Cathodal tDCS over the left and right AC decreased pitch discrimination
Matsushita et al., 2015	42	Right temporal cortex, 35 cm^2^	Above left eyebrow, 35 cm^2^	1.0 mA for 20 min	Anodal cathodal sham	Online and offline	Pitch discrimination task	Anodal tDCS over the right AC decreased pitch discrimination
Schaal et al., 2013	24	CP3, 25 cm^2^	Contralateral supraorbital, 35 cm^2^	2.0 mA for 20 min	Anodal sham	Online and offline	Pitch memory tasks (recognition and recall)	Cathodal tDCS diminished pitch discrimination
Schaal et al., 2015	72	CP3, CP4, 25 cm^2^	Contralateral supraorbital, 35 cm^2^	2.0 mA for 20 min	Cathodal sham	Online and offline	Pitch memory tasks (recognition and recall)	Cathodal tDCS over the left SMG decreased pitch discrimination in non-musicians, cathodal tDCS over the right SMG diminished pitch recognition in musicians
Tang and Hammond, 2013	20	C4-T4, 24 cm^2^	Contralateral supraorbital, 24 cm^2^	1.0 mA for 20 min	Anodal sham	Online	Frequency discrimination task, place coding, temporal coding	Anodal tDCS over the right AC diminished frequency discrimination and sensitivity to temporal fine structure
Vines et al., 2006	11	TP3, TP4, 15 cm^2^	Contralateral supraorbital, 30 cm^2^	1.2 mA for 20 min	Cathodal sham	Offline	Pitch memory task	Anodal tDCS over the left SMG enhanced pitch memory
Zaehle et al., 2011	14	T7, CP5, 35 cm^2^	contralateral supraorbital, 35 cm^2^	1.25 mA for 11 min	Anodal cathodal sham	Offline	Passive listening task, AEPs	Anodal tDCS over temporal lobe enhanced P50 component, cathodal tDCS over the temporo-parietal region enhanced N1 component


*MMN, mismatch negativity; SMG, supramarginal gyrus; AEP, auditory evoked potential; electrode positions refer to the international 10–20 system.*

To modulate individual auditory temporal resolution abilities, Ladeira et al. (2011) used a bilateral auditory tDCS schema with two active electrodes over the AC and a non-cephalic reference electrode over the right deltoid muscle. Such a non-cephalic reference electrode eliminates potential confounding effects of the reference electrode (Ferrucci et al., 2008; Priori et al., 2008). Given that the reference electrode has a polarity opposite to the active electrode, an effect of a cephalic reference location should be necessarily considered because unwanted reversed effects on underlying cortical areas may mislead the interpretation of outcome results. In this study, participants performed an auditory gap detection task while they received bilateral anodal, cathodal, or sham tDCS (online effect). As a result, tDCS caused polarity-dependent alterations of the temporal processing activity of the AC. While bilateral anodal tDCS improved the perceptual performance by up to 22.5%, cathodal stimulation decreased the performance by 54.5%. The data convincingly show that temporal resolution of the AC can be externally modulated by tDCS.

Although this study demonstrated an improvement of temporal resolution abilities after bilateral auditory tDCS, traditionally, the AC has been proposed to show a relative trade-off in spectral and temporal processing of complex acoustic signals such as speech and music, with left auditory cortical areas being tuned for temporal resolution and right auditory cortical areas being more amenable to spectral resolution (Zatorre and Belin, 2001; Poeppel, 2003). Accordingly, to test the hypothesis of a left hemispheric dominance for temporal processing, we applied a lateralized stimulation schema, selectively stimulating either the left or the right AC by anodal tDCS (Heimrath et al., 2014). To increase focal precision and minimize biasing reference effects, we used a small 5 cm × 5 cm active electrode and a larger 5 cm × 10 cm reference electrode placed contralateral over C4/C3 (according to the international 10–20 system). Participants received on three separate days one session of sham, anodal stimulation over the left or over the right AC in a randomized order. After 10 min tDCS, participants performed an auditory temporal resolution task, while stimulation continued until participants finished the task (online effect). Our results showed that neuromodulation of the left, but not right, AC altered individual temporal resolution abilities suggesting a predominance of the left AC for processing rapid temporal acoustic information in non-speech sounds. Remarkably, in this study the tDCS-related increase in cortical excitation of the left AC resulted in deteriorated auditory performance. As mentioned above, this presumed unintuitive result can be related to a non-linear relationship between stimulation effect and resulting behavior. In particular, we assume an inverted U-shaped dose-response relationship between AC reactivity and auditory perception. Although the influence of tDCS on the auditory activity state is possibly a multifactorial phenomenon, the arousal level crucially interacts with perceptual processes and influences the efficiency at a given dosage. The performance improves as arousal increases until it reaches a point where an optimal performance is achieved and arousal is at its optimum level. If arousal increases beyond this point, e.g., due to external electric stimulation, performance will begin to deteriorate. This hypothesis implies that enhanced excitability does not increase performance *per se*. Adapting this assumption, one might further speculate that in deficient auditory processing associated with hypofunctioning of the auditory-related cortex (Gaab et al., 2007; Chobert et al., 2012; Raschle et al., 2014), an enhancement of left AC reactivity will result in an improvement of such perceptual processes. This, in turn might foster potential approaches for a treatment of speech-related pathologies such as dyslexia.

Polarity-specific tDCS effects have also been demonstrated for the alteration of spectral acoustic processing. Anodal tDCS over the left supramarginal gyrus (SMG) enhanced (Schaal et al., 2013), whereas cathodal tDCS diminished the performance in a pitch memory task (offline effect) (Vines et al., 2006). Thus, the systematic stimulation of the left SMG provides further support for the functional relevance of this cortical area for pitch processing by adding causal evidence to former correlative fMRI data that already associated pitch memory with left SMG processing (Gaab et al., 2003). In a further study, Schaal et al. (2015) reported impaired pitch discrimination in non-musicians after cathodal tDCS over the left SMG, whereas cathodal stimulation over the right SMG diminished performance in pitch recognition in musicians (offline effect). The results show a causal distinction between left and right SMG for pitch processing in non-musicians and musicians. Analogously, Mathys et al. (2010) investigated hemispheric specialization in pitch discrimination. The application of tDCS showed that anodal tDCS had no effect, whereas cathodal tDCS over the left and right Heschl’s gyrus decremented pitch discrimination abilities with significantly stronger effect after right Heschl’s gyrus stimulation. Thereby, the authors causally demonstrated that the right Heschl’s gyrus is predominantly involved in pitch discrimination. Moreover, Tang and Hammond (2013) applied anodal tDCS over the right AC and demonstrated diminished frequency discrimination (online effect) further evidencing right-lateralized specialization for spectral processing (Tang and Hammond, 2013). Further confirmation for the importance of the right primary AC in pitch discrimination has recently been provided by Matsushita et al. (2015). The authors showed that tDCS over the right Heschl’s gyrus with the reference over the left eyebrow affected pitch discrimination performance (online effect). Interestingly, while cathodal tDCS had no effect, anodal tDCS interrupted performance. Finally, tDCS of the auditory fronto-temporal network also influences spectral acoustic processing. Loui et al. (2010) demonstrated decreased performance in a pitch matching task after cathodal tDCS over the left posterior inferior frontal gyrus (IFG) and right superior temporal gyrus indicating a causal role of the fronto-temporal network for pitch processing.

Overall results show that tDCS application over the AC changes its neuronal reactivity and thereby systematically induces up- or downregulation of acoustic processing. Even though some reported data show a polarity-specific effect on behavior, there are also reports on opposite tDCS effects further questioning the assumption that changes of cortical excitability are simply reflected in behavioral effects (Jacobson et al., 2012; Miniussi and Ruzzoli, 2013). Consequently, the investigation of tES induced neuronal effects requires direct electrophysiological assessment by EEG. Further problems associated with these variable results are varying task demands and task dependent attention effects that can systematically cause behavioral variations (Bryden et al., 1983; Woldorff et al., 1993; Poeppel et al., 1996). Accordingly, it is necessary to minimize these confounds or to separate tDCS effects of interest (e.g., perception) from effects on attentional or motivational factors. To specifically address this issue, acoustic perception without attentional demands can be assessed by recording the mismatch negativity (MMN), a pre-attentive measure of event-related potentials (Kujala et al., 2007). MMN occurs as a negative component that can be elicited by infrequently occurring deviant tones in a sequence of frequently occurring standard tones. Auditory MMN has been assumed to originate bilaterally in the supratemporal and auditory cortices (Naatanen et al., 2007, 2011) as well as the prefrontal cortex (Deouell, 2007; Garrido et al., 2009). Accordingly, the MMN allows for the acquisition of direct electrophysiological consequences of auditory tDCS without confounding cognitive influences.

Consequently, in a recent study, MMNs to spectral deviants were assessed after anodal and sham tDCS were applied over the left AC (Impey and Knott, 2015). They demonstrated that anodal tDCS enhanced spectral deviance processing, but only in individuals with low MMN baseline amplitudes, whereas subjects with higher baseline deviant detection abilities showed no tDCS related MMN amplitude increase (offline effect). The authors argued that anodal tDCS on subjects with high deviance detection ability is less effective due to ceiling effects and only low performers may benefit from electrical stimulation. However, spectral deviance processing could not only be improved by anodal tDCS of the left AC, but also by anodal tDCS over the right frontal cortex. Chen et al. (2013) assessed the influence of anodal, cathodal, and sham tDCS delivered over the right inferior frontal cortex on MMN in response to temporal and spectral auditory deviants. They showed that anodal tDCS over the right frontal cortex exclusively decreased MMN amplitude to spectral deviants whereas neither anodal nor cathodal tDCS modulated MMN to temporal deviants (offline effect). The results show that each part of the underlying fronto-temporal cortical network can be influenced by tDCS resulting in alterations of pre-attentive deviance detection assessed via auditory MMN measurement.

To further test functional lateralization in the human auditory system, we assessed the influence of anodal and cathodal high-definition (HD)-tDCS delivered over the left or right AC on auditory MMN in response to temporal as well as spectral deviants (Heimrath et al., 2015). Computational modeling of current density provides evidence that conventional tDCS with relatively large electrode-pads (25cm^2^ – 35cm^2^) stimulates rather broad cortical areas. To improve the spatial preciseness, HD-tDCS has been introduced (Datta et al., 2009). In contrast to conventional tDCS for HD-tDCS a 4 × 1 ring configuration with a center electrode overlying the targeted brain area surrounded by four reference electrodes enables a more restricted cortical neuromodulation (Kuo et al., 2013), higher electric fields in comparison to electrode pads (Datta et al., 2012) and minimization of the confounding effect of a single reference electrode. Moreover, such a stimulation schema over auditory cortical areas allows for parallel EEG recording at central electrodes. Thus, by applying HD-tDCS of the AC electrophysiological recordings can be acquired directly during stimulation (online effect) without relying on transient offline effects (Heimrath et al., 2015). In this study, we applied a central active electrode over the left and right AC surrounded by four reference electrodes. While receiving anodal or cathodal HD-tDCS participants performed an auditory MMN paradigm (Naatanen et al., 2007) with spectral and temporal deviants. Specifically, after 10 min of consecutive HD-tDCS the MMN paradigm started while the stimulation continued. The results showed that MMN amplitude in response to temporal but not spectral acoustic features was elevated during anodal HD-tDCS of the left AC only (Heimrath et al., 2015). Cathodal tDCS over the left and right AC had no effect on MMN amplitude to neither spectral nor temporal deviants. Consequently, our data provide causal evidence for a left hemispheric dominance for the pre-attentive processing of low-level temporal information. To our knowledge this was the first multimodal approach applying electrophysiological recordings during auditory tDCS to gain more detailed information about the underlying neuronal mechanisms involved in these alterations.

While several studies investigated the influence of tDCS on low-level acoustic processing only, less is known about the efficiency of auditory tDCS on speech perception. In a recent study we investigated the modulation of acoustic speech perception in a phonetic categorization task by bilateral auditory tDCS. For the bilateral stimulation we attached two small 5 cm × 5 cm active electrodes over the left and right AC and a larger 5 cm × 10 cm electrode longitudinally over central sites. After 10 min of anodal or cathodal tDCS a phonetic categorization task started while stimulation continued. In this task, participants were presented with a synthetic voice onset time (VOT) continuum ranging from 20 to 40 ms VOT in 1 ms steps and had to decide whether each presented syllable was the voiced syllable /da/ or the voiceless syllable /ta/. We found that online cathodal tDCS improved phonetic categorization abilities. In fact, concurrent cathodal tDCS steepened the slope of the identification curve indicating more precise categorization of the syllables /ta/ and /da/. In a subsequent (offline) assessment of the neurophysiological changes after stimulation, participants were presented with canonical voiced and voiceless syllables while their EEG was measured. Again, replicating former results (Zaehle et al., 2011) the P50 amplitude of the AEP to all syllables was selectively elevated after anodal tDCS (Heimrath et al., 2016). Thus uni- as well as bilateral anodal tDCS over the primary auditory region increases P50 amplitudes to acoustic stimuli. Since P50 presumably reflects sensory representation of an acoustic stimulus in the AC (Sharma et al., 2002; Ceponiene et al., 2009), we provide direct electrophysiological evidence for a tDCS related modulation of sensory phoneme processing.

In general, the data show that tDCS application, separately, and in combination with EEG, constitutes a promising approach to investigate and modulate neuronal activity involved in acoustic processing. Consequently, tDCS may serve as a clinical tool for the treatment of AC related disorders observed in tinnitus or aphasic patients.

## Effects of tACS/tRNS on Endogenous Oscillations in the Auditory System

The sequential series of action potentials in the human cortex can be measured as oscillatory patterns both intracranially and extracranially, the latter by use of EEG or MEG. Over the last years, numerous studies convincingly showed that such neural oscillations are not mere epiphenomena. In concrete terms, specific features of brain oscillations, such as amplitude, frequency and phase are causally linked to brain functions and have been shown to be fundamental in perception, cognition, and learning. While slower oscillations (i.e., in the theta band around 4–8 Hz) are associated with long-range cortico-cortical information transfer, faster oscillations (i.e., in the gamma band around 30–80 Hz) represent a more local information transfer in neural ensembles. This information transfer is represented by synchronization of the oscillatory frequency of two or more involved cortical regions (Buzsaki and Draguhn, 2004).

Oscillatory activation patterns are also fundamental in auditory perception (Lakatos et al., 2005, 2008; Schroeder and Lakatos, 2009) and altered oscillation patterns have been associated with impaired processing of auditory stimuli. Consequently, modulation of these inherent oscillation patterns (e.g., via external stimulation) should facilitate auditory perception and, more importantly, should also improve diminished auditory processing in neuropsychological disorders.

While on the behavioral level it has been demonstrated that transcranially applied alternating current can modulate visual perception (Kanai et al., 2008), and motor performance (Pogosyan et al., 2009), we directly evidenced tACS induced neuronal oscillatory entrainment in an early study (Zaehle et al., 2010). After applying 10 min tACS at the individual alpha frequency (IAF) over parietal cortex regions we demonstrated increased power in the alpha band (offline effect). While these initial studies supported the general notion of tACS as a valid tool to modulate behavior by increasing the oscillatory coherence in the relevant cortical areas, there is still sparse knowledge about the effect of tACS on auditory processing. Due to the lack of systematic investigations of the impact of stimulation parameters such as the applied current, electrode size, and stimulation frequency so far no standard paradigm for improving performance has been established.

In a first approach, Neuling et al. (2012a) investigated the influence of 10 Hz tACS over the AC on auditory perception. Subjects had to detect sine wave tones embedded in white noise of different signal-to-noise ratio while sham or verum tACS was applied over the bilateral AC region. Stimuli were presented at different time points relative to the phase angle of the 10 Hz tAC-stimulation. The authors found increased alpha band power after tACS relative to the pre-stimulation period (offline effect). While they generally confirmed our earlier findings of tACS induced neuronal entrainment (Zaehle et al., 2010), they additionally showed that this entrainment was also evident in the AC region. Moreover, the study demonstrates that the acoustic precision varied depending on the phase of the applied tACS oscillation at which the stimuli were presented. When the stimuli were presented in the phase range of 0–180° individual detection rates were significantly enhanced compared to when stimuli were presented at phase angles of 181–360° (online effect). In sum, this study reveals that 10 Hz tACS increases alpha power in the AC regions and modulates auditory sensitivity.

Besides the application of sinusoidal tACS in the alpha range, slower delta/theta (4 Hz) sinusoidal electric stimulation can also modify auditory perception (Riecke et al., 2015). While participants received 4 Hz tACS over bilateral temporal cortex they performed an auditory detection task. Again, individual detection rates significantly correlated with the phase angle of the induced 4 Hz oscillations. Subjects’ performance was above baseline when click trains were presented during the phase range of 0–180°, whereas it was below baseline when presented in the phase range of 181–360° (online effect).

Accordingly, these findings consistently demonstrate that sinusoidal tACS is able to entrain endogenous oscillations in the AC. Furthermore, acoustic sensitivity seems to depend on the phase of these neuronal oscillations at which the auditory stimulus is presented.

Focusing on more speech-related processes, we investigated the functional relevance of gamma oscillations in auditory phoneme processing (Rufener et al., 2016). Previous studies assessing the neural mechanism underlying successful speech processing emphasized the functional relevance of different endogenous oscillation patterns with theta oscillations (4–8 Hz) being more important for the processing of intonation contour and gamma oscillation (30–48 Hz) being essential in phoneme processing (Poeppel, 2003; Giraud and Poeppel, 2012; Pena and Melloni, 2012; Gross et al., 2013). To causally test the assumption of such a close functional relationship between AC gamma oscillations and phoneme processing, we assessed the influence of sham, 6 and 40 Hz tACS on subjects’ phonetic categorization ability. Presented with artificially manipulated sounds ranging from the phonemes /da/ to /ta/ subjects had to indicate for each stimulus whether they perceived the initial consonants as voiced or voiceless. Subjects performed this task in five consecutive runs: pre-tACS, during three runs of tACS over bilateral AC region, as well as in a post-tACS run. Comparing pre-to-post categorization accuracy, 40 Hz tACS selectively decreased performance compared to sham and 6 Hz tACS, causally confirming the functional relevance of 40 Hz oscillations in phoneme processing (offline effect).

At the interface between auditory perception and memory functions, Schaal et al. (2015) assessed the influence of tACS in a sample of amusic patients. Amusia, a congenital neuropsychological disorder, characterized by pitch perception deficits and impaired pitch memory, is caused by structural and functional impairments including a reduced connectivity between frontal and temporal areas in the right hemisphere. Amusia has also been associated with deficits in language perception and intonation perception (Hyde et al., 2009; Loui et al., 2009; Liu et al., 2010), accompanied by reduced gamma oscillations in the right dorsolateral prefrontal cortex (DLPFC). Therefore, the authors applied 35 Hz tACS over the right DLPFC and contra-lateral supraorbital area while participants performed an auditory memory task (online effect). By evidencing that 35 Hz tACS can increase auditory memory in amusic patients, the study demonstrates that the applicability of tACS is not limited to auditory perception at the level of the AC. Rather, the study highlights that tACS can modulate neural processes at different hierarchical levels.

In a first proof-of-principle study, Terney et al. (2008) demonstrated the efficacy of tRNS to enhance motor cortex excitability that lasted up to 60 min post stimulation (online effect). In addition, they found a superior effect of high frequency tRNS (101–640 Hz) compared to low frequency tRNS (0.1–100 Hz). The effect of tRNS on AC reactivity has only been assessed in one study so far. Van Doren et al. (2014) investigated tRNS related modulations of auditory steady-state responses (ASSRs). In this study, high frequency tRNS induced an increase in power at 40 Hz ASSR, demonstrating the ability of tRNS to modulate information processing in the vicinity of the AC (offline effect).

Taken together, the current state of research using tES provides evidence for the applicability of tES over the AC. Despite the distinctive heterogeneity in the tES procedure, the reviewed studies show convincingly that tES can modulate acoustic perception as well as both the general reactivity of the AC and the synchronization of neural oscillations in the vicinity of the AC. These effects were present on a behavioral level as well as in neurophysiological parameters. Accordingly, processing of auditory information in healthy subjects can be systematically modulated by means of tES. The stimulation parameters and outcome measures of the cited studies are summarized in **Table 2**.

**Table 2:** General parameters and results of tACS/tRNS studies.

Reference	Sample size	Electrode position and size	Stimulation parameters	Stimulation frequency	Timepoint of data acquisition	Paradigm	Results
Neuling et al., 2012a	20	T7 and T8, 35 cm^2^	Below individual sensation threshold for 21 min	Oscillatory tDCS (10 Hz)	Online	Auditory threshold detection	Phase dependent detection thresholds; improved performance with stimulus presentation at 0–180° and hampered performance at 180–360°
Riecke et al., 2015	14	T7 and T8, 25 cm^2^ and left and right to Cz, 35 cm^2^	Below individual sensation threshold for 40 min	4 Hz	Online	Auditory threshold detection	tACS phase dependent detection thresholds; improved performance with stimulus presentation at 0–180° and hampered performance at 180–360°
Rufener et al., 2016	35	T7 and T8, 35 cm^2^	Below individual sensation threshold for 20 min	40 Hz, 6 Hz	Offline	VOT-categorization	40 Hz tACS impaired VOT-categorization ability
Schaal et al., 2015	17	Right DLPFC and contralateral orbit, 25 cm^2^	1 mA for 20 min	35 Hz, 90 Hz	Offline	Pitch memory	35 Hz tACS facilitated pitch memory, leading to comparable performance in congenital amusics and controls
Van Doren et al., 2014	14	T7 and T8, 35 cm^2^	2 mA for 20 min	tRNS (100–640 Hz)	Offline	Auditory steady state response (ASSR)	Increased ASSR-response to 40 Hz frequency-modulated tone


*VOT, voice onset time; DLPFC, dorsolateral prefrontal cortex; electrode positions refer to the international 10–20 system.*

## Clinical Approaches for Auditory Related Disorders

Even though there is still relatively little known about the underlying neurophysiological mechanisms of auditory tES, therapeutic stimulation effects have been explored in various neurological populations with auditory related disorders. Given the neuromodulatory potential of these techniques there are several studies investigating tES as an alternative therapy approach beyond behavioral treatment. In the following, we will concentrate on tinnitus and post-stroke aphasia as two major challenges in neurorehabilitation.

Tinnitus is a widely distributed disease of the central auditory system proposed to originate from plastic changes and hyperactivity in auditory and non-auditory brain structures (Muhlnickel et al., 1998; Salvi et al., 2000; Langguth and De Ridder, 2013). In the current literature the most common cortical targets for tES are the TPA and the DLPFC. These regions have been consistently associated with auditory processing and are presumed to be involved in tinnitus pathogenesis (Mirz et al., 2000; Schlee et al., 2009). Within the TPA electrical stimulation is applied to modulate activity in the primary AC as well as auditory association areas (Shekhawat et al., 2015c). Studies indicated that the DLPFC plays a role in auditory memory (Bodner et al., 1996; Alain et al., 1998) and has been associated with auditory attention (Voisin et al., 2006). The DLPFC exerts inhibitory control of input to primary auditory regions (Knight et al., 1989), leading to a top–down modulation of acoustic processing (Mitchell et al., 2005). Dysfunctions in these prefrontal inhibitory processes might lead to tinnitus symptoms (Norena et al., 1999).

Multiple studies provided evidence for beneficial effects of anodal tDCS over the right DLPFC or left TPA on subjectively rated transient tinnitus loudness and annoyance (Fregni et al., 2006; Garin et al., 2011; De Ridder and Vanneste, 2012; Faber et al., 2012; Shekhawat et al., 2013b, 2015c; Joos et al., 2014). Cathodal stimulation of these brain areas failed to induce significant effects (Vanneste et al., 2010; Garin et al., 2011; Joos et al., 2014). Interestingly, a systematic comparison of different stimulation sites by Shekhawat et al. (2015c) demonstrated that anodal stimulation of either the left TPA or the right DLPFC are equally effective for subjective tinnitus relief. Furthermore, Shekhawat et al. (2013b) investigated the effects of different stimulation parameters for the application of anodal tDCS over the left TPA and revealed that a current intensity of 2 mA delivered for 20 min is the most effective setting, leading to a transient subjective tinnitus suppression in 56% of participants.

Importantly, for clinical application the achievement of persistent rather than acute symptomatic improvements is highly relevant. While the majority of studies did not test for long-term effects, in the study of Garin et al. (2011) half of the patients reported longer lasting effects of tDCS, some persisting more than 2 weeks after stimulation. However, the aftereffects were heterogeneous with some patients declaring a tinnitus reduction, while others reported a worsening of tinnitus symptoms. In contrast to these findings, other studies failed to show tinnitus suppressive effects of tDCS applied over the TPA or DLPFC (Shekhawat et al., 2013a, 2015a; Teismann et al., 2014; Cavalcanti et al., 2015; Pal et al., 2015). When combining tDCS with standard behavioral treatments like music training or hearing aids, improvement in tinnitus appeared independent of tDCS condition (Shekhawat et al., 2013a; Teismann et al., 2014). Interestingly, in all of these studies patients underwent four or five consecutive tDCS sessions. Accordingly, Cavalcanti et al. (2015) argued that stronger improvements in tinnitus symptoms may only be measurable after a first, single tDCS session before a process of habituation might occur. Furthermore, Pal et al. (2015) targeted multiple stimulation sites simultaneously with two cathodes overlying the AC bilaterally and the anode over the prefrontal cortex. As previous studies targeting either the TPA or the DLPFC already revealed tinnitus suppressive effects, the efficacy of different stimulation protocols needs to be considered.

Importantly, Vanneste et al. (2013) compared the efficacy of tDCS, tACS, and tRNS in tinnitus suppression. By applying a current intensity of 1.5 mA for 20 min over the bilateral AC, both tDCS, and tACS failed to induce significant effects. Given beneficial effects of single-sided tDCS of the AC (Fregni et al., 2006; Garin et al., 2011; Shekhawat et al., 2013b; Joos et al., 2014), a bilateral stimulation might not be the optimal electrode montage. Interestingly, only tRNS induced transient improvements in tinnitus loudness and distress. Previous studies proposed a pathological hyper-synchronization within the AC of tinnitus patients (Weisz et al., 2007; Tass et al., 2012). Therefore adding random noise by means of tRNS might disrupt this synchronization resulting in tinnitus suppression (Vanneste et al., 2013). This is in line with latter studies providing evidence for tinnitus relief by tRNS (Claes et al., 2014; Joos et al., 2015).

Overall, the current literature reveals beneficial effects of tRNS of the bilateral AC as well as anodal tDCS over the right DLPFC or left TPA on tinnitus symptoms. However, the high interindividual variability of treatment effects requires prospective investigations by randomized clinical trials with larger sample sizes and optimized stimulation parameters (Langguth and De Ridder, 2013). Furthermore, as most studies focused on transient tinnitus improvements little is known about the persistence of stimulation aftereffects. The assessment of symptom change is further complicated by the dependence on subjective questionnaires due to a lack of objective measures for tinnitus reduction. Finally, as the involvement of multiple brain areas, in particular the TPA and DLPFC (Shekhawat et al., 2015c), in tinnitus hampers the prediction of tES related changes, a combination of stimulation and neuroimaging techniques such as EEG or fMRI can be useful to explore the physiological basis of tES for the treatment of tinnitus (Vanneste and De Ridder, 2011; Vanneste et al., 2011; De Ridder and Vanneste, 2012; Shekhawat et al., 2015b).

Whereas tinnitus is associated with abnormal activity in the auditory system leading to phantom acoustic sensations, acquired brain injury due to stroke or traumatic brain injury can lead to deterioration of the auditory system causing complex language processing deficits. Accordingly, the potential of tES to modulate brain excitability in the auditory network has recently been used in post-stroke aphasia rehabilitation. As conventional speech and language therapy strategies have limited and variable effects (Brady et al., 2012), tES has a potential as a supplementary treatment approach. Generally aphasia recovery depends on neuroplastic changes in the language network, involving reorganization of lesioned and perilesional regions in the language-dominant left hemisphere as well as altered activation in homologous right hemispheric areas (Hamilton et al., 2011; Shah et al., 2013). It has been proposed that following left hemispheric stroke, disinhibited language areas in the right hemisphere may exert an increased inhibitory influence on perilesional regions, limiting language recovery in the left hemisphere (Otal et al., 2015). To restore the balance in interhemispheric inhibition most studies applied left hemispheric anodal tDCS to increase excitability of perilesional areas (Monti et al., 2008; Baker et al., 2010; Fridriksson et al., 2011; Marangolo et al., 2011, 2013) or cathodal tDCS over the right hemisphere to suppress overactivation in contralesional regions (Floel et al., 2011; Jung et al., 2011; Kang et al., 2011), both resulting in improved language performance. Fridriksson et al. (2011) delivered anodal tDCS over left posterior perilesional brain regions during five sessions of a behavioral language treatment. Results revealed an improvement in naming reaction times that persisted at least 3 weeks after stimulation. In contrast to this finding, Monti et al. (2008) demonstrated an improvement in picture naming accuracy after cathodal tDCS over left frontotemporal areas whereas anodal stimulation failed to induce effects. Contradictory polarity effects have also been reported for tDCS applied over right hemispheric language structures (Floel et al., 2011; Jung et al., 2011; Kang et al., 2011). Interestingly, a recent study by Lee et al. (2013) demonstrated that simultaneous application of anodal tDCS over the left IFG and cathodal tDCS over the right IFG might be superior to single anodal tDCS in improving performance in a picture naming test.

Systematic investigations of the persistence of stimulation effects revealed tDCS induced language improvements that lasted up to 2 months (Marangolo et al., 2011) or even up to 16 weeks after treatment (Vestito et al., 2014). Whereas the majority of studies focused on chronic aphasia recovery, there is also evidence for beneficial effects of tDCS in acute aphasia during early stages after stroke (You et al., 2011; Polanowska et al., 2013b). Thus, the time window for effective tDCS treatment is an important issue to consider.

In sum, the current literature shows controversial results regarding the effectiveness of tES in aphasia recovery with some studies demonstrating improvements in language performance whereas others revealed no benefits of stimulation (Polanowska et al., 2013a; Elsner et al., 2015). As the therapeutic effects of tDCS may vary for different types of aphasia and individual patient characteristics (Shah-Basak et al., 2015), optimal stimulation parameters remain to be determined. Accordingly, systematic investigations on stimulation side, polarity, duration, current density of stimulation and the frequency of stimulation sessions are required (Shah et al., 2013). Despite the described heterogeneities, the reported studies show encouraging results for the use of tDCS in neurorehabilitation of the auditory language network.

## Outline and Future Perspectives

Notwithstanding the neurophysiological mechanisms underlying tES induced behavioral and physiological alterations are still not fully understood, the current findings show that tES can alter auditory perceptual processing and consequently constitutes a clinical tool for the treatment of auditory related disorders. In the following section, current results of tES will be discussed in the light of possibilities, pitfalls, as well as prospective clinical application in dyslexia, which is one of the most frequently diagnosed neuropsychological disorders affecting auditory and language processing.

Dyslexia is a learning disorder characterized by severe and persistent reading and spelling problems. The prevalence of dyslexia has been estimated to be approximately 5 to 10% (Elliott and Grigorenko, 2014). Despite the fact that the current focus of research lies on child and adolescent dyslexics, most affected subjects report persistent restrictions in reading and writing in adulthood. One of the most dominant cognitive symptoms of dyslexia is the phonological processing deficit. The impaired phonological skills are the consequence of a more basic auditory processing constraint that disrupts essential components for literacy, starting with the acquisition of phonological representations (Ramus, 2003; Tallal, 2004; Tallal and Gaab, 2006). At the neurological level, the perceptual deficit is related to a dysfunction of left hemispheric perisylvian brain areas that underlie phonological representations (Ramus, 2003). The impaired auditory processing impedes speech perception by degrading the ability to accurately segment the speech stream into its important phonetic components such as rhymes, syllables, and phonemes. Accordingly, individuals with dyslexia have difficulties in processing rapidly changing information in speech – such as the spectral changes of formant transitions (Farmer and Klein, 1995; Tallal and Gaab, 2006) and cues that vary over time such as amplitude and frequency modulations (Studdert-Kennedy and Mody, 1995) – as well as in non-speech sounds (Tallal and Piercy, 1974; Breier et al., 2001; Chandrasekaran et al., 2009). Accordingly, basic auditory processing problems can be considered to be causally responsible for phonological deficits (Farmer and Klein, 1995; Tallal, 2004; Tallal and Gaab, 2006). Given the fact, that the vast majority of dyslexic patients shows a deficit in low-level auditory temporal processing both of speech-specific stimuli (Merzenich et al., 1993; Gaab et al., 2007; Vandermosten et al., 2010; Raschle et al., 2014) as well as of non-speech stimuli (Tallal and Piercy, 1974; Breier et al., 2001; Chandrasekaran et al., 2009) and the evidence of tDCS associated alterations of basic auditory performance reviewed above, the application of tDCS seems to be a promising technique to improve both the AC reactivity in dyslexics and the impaired processing of incoming speech features.

Moreover, deficient acoustic processing abilities in dyslexia have also been associated with altered oscillatory patterns related to profound structural and functional alterations in the bilateral perisylvian areas (Hutchison et al., 2008; Lehongre et al., 2011, 2013; Kovelman et al., 2012). Given the potential of tACS to entrain neural oscillations in the AC at various frequencies the applicability of tACS to restore altered oscillation patterns in dyslexics seems feasible. Following this, one might speculate that tACS induced restoration of deteriorated neural mechanisms would result in enhanced auditory processing ability and, in turn, in improved linguistic abilities such as reading and writing.

However, although there is convincing evidence on the applicability and the potentially beneficial effect of tES in dyslexic samples a number of important prerequisites have to be taken into account in order to successfully utilize tES in clinical samples. In the following section, we will discuss some of the most important methodological pitfalls.

### Methodological Challenges of tES

**FIGURE 2:**
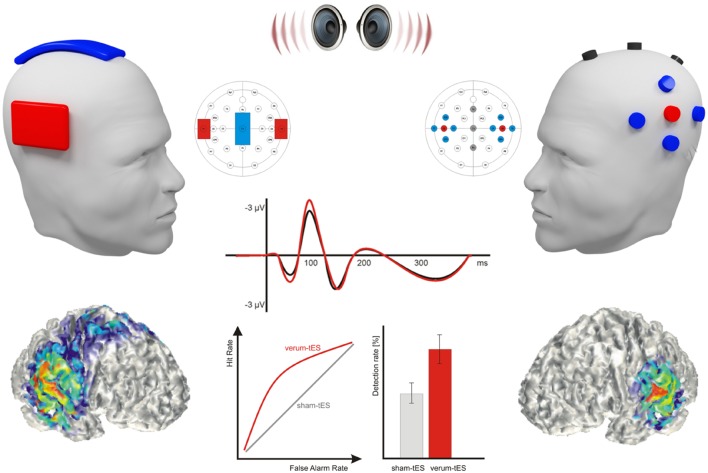
**Illustration of the auditory tES effects.** Figure exemplifies two different electrode montage schemas: conventional tES (left) with active sponge-pad electrodes over the target areas and a larger reference electrode over the vertex and 4 × 1 High-Definition (HD)-tES (right) with central active ring electrodes surrounded by four reference electrodes. While the conventional tES schema enables the online assessment of behavioral effects only, HD-tES additionally allows for the online assessment of EEG from midline electrodes (e.g., Fz, Cz, Pz). Both tES schemas induce current density maxima at the auditory cortices (AC) leading to alterations of the neuronal reactivity within the stimulated area. As illustrated below, whereas conventional tES innervates rather broad cortical areas, HD-tES is supposed to allow for a more focal stimulation. In consequence auditory tES results in changes in the electrophysiological reactivity to auditory stimulation [such as altered P50-N1 amplitudes of the auditory evoked potential (AEPs)] and in corresponding alterations in auditory task performance (e.g., enhanced perceptual sensitivity or detection rate).

Generally, stimulation parameters such as stimulation intensity, electrode size, electrode placement especially of the reference electrode as well as interindividual variability of subjects need to be systematically investigated for the implementation of optimal tES protocols on the auditory system (for an overview on parameters of previous studies see **Tables 1** and **2**). One main challenge in future research and clinical application of tES is the improvement of stimulation focality. HD-tDCS has been recently advanced to overcome this issue by utilizing small ring electrodes that increase spatial specificity of the current over the targeted cortical area (Datta et al., 2009; Heimrath et al., 2015) (see **Figure 2**).

However, auditory processing is a complex cognitive function that is mediated by multiple functionally connected brain areas rather than one individual region, which is typically targeted in tES studies (Vanneste and De Ridder, 2011). TES over, e.g., the perisylvian region does not only modulate brain activity in the region under the electrode but also in other functionally related areas (Wu et al., 2015). Accordingly, a combination of tES and neuroimaging techniques is recommended to explore the connections between different areas of the auditory network and to understand whether and how tES may influence network excitability. This might further enable the development of efficient tES protocols to target specific connections between brain areas within the auditory network (Luft et al., 2014).

Importantly, most of the reported studies investigated tES online effects on auditory perception only, while electrophysiological investigations of tES online effects are sparse. Due to the strong artifacts induced by tES, such electrophysiological data were only measurable offline, after terminating the stimulation. Thus, the effect of tES on the underlying neural mechanism remains an important, but yet fairly under investigated question. Further knowledge about tES effects on brain functions may improve the specificity of stimulation protocols for clinical samples with auditory processing disorders. While several studies reporting pre-to-post changes in event-related potentials or resting-EEG data used offline measurements, to date, there are only a limited number of studies combining tES and electrophysiological data. It has been suggested that MEG is able to overcome this shortcoming due to its measurement of neuromagnetic activity via sensors [i.e., superconducting quantum interference devices, (SQUIDS)] not directly placed at the scalp (Soekadar et al., 2013; Neuling et al., 2015; Witkowski et al., in press). The use of HD-tES electrode application might be a further promising approach for the parallel assessment of EEG during tES. Such HD-tES in combination with simultaneous EEG acquisition has been successfully demonstrated for the HD-tDCS modulation of the AC reactivity (Heimrath et al., 2015) as well as tACS induced entrainment of endogenous alpha oscillations (Helfrich et al., 2014).

Finally, besides the contributions of stimulation parameters and task difficulty on the variability of response to tES both inter-individuals and across multiple testing sessions, also baseline activity changes within the targeted neural network have to be considered. In particular, rather than exerting a homogeneous effect on each neuron underneath the electrodes and across individuals, tES instead interacts with endogenous activity levels within target neuronal populations. This results in tES outcomes that are dependent on the pre-existing activation state of the targeted neurons at the moment of stimulation, i.e., on baseline activity (Krause et al., 2013). Again, further research particularly combining tES and direct neurophysiological measures of brain activity are necessary to further examine the relationship between neuronal baseline activity and effects of stimulation.

### Specific Prerequisites of tES for Clinical Application

The vast majority of all clinical interventions target to normalize pathological processes and to ensure the prolonged impact of the completed intervention. Thus, in order to use tES in a clinical setting it is vital to know whether the stimulation schema results in aftereffects of adequate duration. Yet there are no systematic investigations on tES long-term effects in the auditory domain.

It is well documented that tDCS over the motor cortex can induce excitability changes from minutes to hours (offline effect). In contrast to transient aftereffects (offline effect), long-term effects persisting over a prolonged period (i.e., days and months) are crucial for the clinical application of tES. In patients with dyscalculia a 6-days training accompanied by anodal tDCS enhanced whereas cathodal tDCS diminished the numerical proficiency. The improvement of performance lasted up to 6 months after the training (Cohen Kadosh et al., 2010). Moreover, five consecutive days of arithmetic training accompanied by tRNS over bilateral DLPFC induced positive effects both on behavior and the neurophysiological level that lasted up to 6 months after the intervention (Snowball et al., 2013). Finally, clinical trials with patients suffering from auditory related disorders show sustained improvement of symptoms lasting up to 16 weeks after the tDCS session (Fridriksson et al., 2011; Garin et al., 2011; Marangolo et al., 2011; Frank et al., 2012; Vestito et al., 2014). Thus, tDCS, especially when applied repetitively on consecutive days seems to be able to induce clinically relevant long-term effects. By contrast, little is known about long-term effects of tACS.

So far, only few tACS studies evidenced sustained aftereffects, a prerequisite for potential long-term effects. A single 20-min session of 10 Hz tACS over the bilateral AC induced sustained aftereffects for up to 30 min (Neuling et al., 2012a, 2013), while one second-episodes of alpha tACS were inefficient at evoking measurable aftereffects (Struber et al., 2015). For the application of tACS at the IAF over the occipito-parietal cortex region, periods of three seconds were not effective in inducing aftereffects whereas eight seconds of IAF tACS led to sustained aftereffects (Vossen et al., 2015). Importantly, the data provide strong evidence that the aftereffects of tACS do not reflect ongoing echoes of the stimulation but that they rather represent neuroplastic changes at the cell level evoked by the stimulation. However, it remains an open question whether (1) the aftereffect would have been measurable with a more sensitive technique (i.e., intracranial electrodes), (2) the missing aftereffect resulted from the chosen tACS parameters (frequency, current intensity, cortical areas stimulated), or (3) one second of tACS is just insufficient to evoke sustained aftereffects.

Finally, for the clinical application of tES, it is desirable to use tES already in early stages of the disorder, when the brain’s ability to adapt to external events and to develop novel strategies is most pronounced. While the application of tES usually aims to improve perceptual or cognitive abilities it seems important to consider probable unwanted side effects in the vulnerable child’s and adolescent’s brain. Despite first promising results in pediatric samples there is still a lack of systematic studies on the effect of tES on the developing brain.

In order to assess both the effect and tolerability of tDCS in the developing brain, Moliadze et al. (2015) investigated immediate effects as well as aftereffects on the excitability of the motor cortex in a sample of children and adolescents (aged 11–16 years). Neither anodal tDCS nor cathodal tDCS with 1 mA for 10 min showed any adverse effects or pathological neuronal activity. In a further study, they reported increased motor evoked potentials to be observed up to one hour after 1 mA anodal and cathodal tDCS (Moliadze et al., 2015). In sum, all children and adolescents tolerated the stimulation well and tDCS consistently induced functional changes in this pediatric sample. None of the participants reported any visual sensations, headache or symptoms of hyperactivity. This, in turn, seems to allow for the application in clinical interventions. Accordingly the clinical efficacy of tDCS in adolescent patients with attention deficit hyperactivity disorder has already been successfully demonstrated (Munz et al., 2015).

Taken together, the use of tES in vulnerable persons and especially children should always be performed with great caution, taking into account all the relevant factors which might have immediate as well as long-term unwanted side effects. Nevertheless, the reviewed articles demonstrated the safety and tolerability of tES in pediatric samples as well as the ability of tES to improve symptoms in adolescent clinical populations. The current results are promising and indicate the importance to force investigations on stimulation parameters in order to develop successful tES protocols for therapeutic application.

## Author Contributions

Wrote the paper: KH, MF, KR, TZ.

## Conflict of Interest Statement

The authors declare that the research was conducted in the absence of any commercial or financial relationships that could be construed as a potential conflict of interest.
